# T236. TECHNOLOGY LITERACY AND ENGAGEMENT IN PEOPLE WITH SCHIZOPHRENIA

**DOI:** 10.1093/schbul/sby016.512

**Published:** 2018-04-01

**Authors:** Kwok Tung Gordon Wong, Daniel King, Ryan Balzan, Dennis Liu, Cherrie Galletly

**Affiliations:** 1 Northern Adelaide Local Health Network; 2 University of Adelaide; 3 Flinders University; 4 Northern Adelaide Local Health Network, University of Adelaide

## Abstract

**Background:**

Information and interventions for mental illness are increasingly being provided on-line. There is an expectation that citizens have access to the internet and are competent in using technology. People with schizophrenia are often excluded from social engagement, have cognitive impairment and have very limited income; all of which may reduce their use of technology.

This project aimed to assess the use of technology and the internet in people with schizophrenia or schizoaffective disorder, living in the community.

**Methods:**

Face-to-face structured interviews were used to evaluate technology literacy, attitudes towards technology, and access and engagement with technology in 50 people with schizophrenia or schizoaffective disorder aged 18–65 years, living in the community.

**Results:**

About half of the study population had access to a computer, and half had access to the internet at home. Participants’ most common uses of technology were voice-calling, messaging, surfing the internet and accessing Facebook. The use of more advanced functions of technology (calendar, banking, news, health information) were rare. The general attitude among participants was that technology was not a significant part of their lives.

**Discussion:**

Technology literacy and internet access were limited in this population. This needs to be addressed before the on-line delivery of educational information, service information and e-health interventions can be widely utilised in people with schizophrenia.

